# Case Report: A Novel NKX2-5 Mutation in a Family With Congenital Heart Defects, Left Ventricular Non-compaction, Conduction Disease, and Sudden Cardiac Death

**DOI:** 10.3389/fcvm.2021.691203

**Published:** 2021-07-01

**Authors:** Paula Morlanes-Gracia, Guido Antoniutti, Jorge Alvarez-Rubio, Laura Torres-Juan, Damian Heine-Suñer, Tomás Ripoll-Vera

**Affiliations:** ^1^Hospital Clínico Lozano Blesa, Zaragoza, Spain; ^2^Hospital Universitario Son LLàtzer, Palma de Mallorca, Spain; ^3^Instituto de Investigación Sanitaria Illes Balears (IdISBa), Palma de Mallorca, Spain; ^4^Departmento de Genetica Clínica y Molecular, Hospital Universitario Son Espases, Palma de Mallorca, Spain; ^5^Centro de Investigación Biomédica CIBEROBN, Madrid, Spain

**Keywords:** NKX2-5, genetic, congenital heart disease, sudden cardiac death, left ventricular non-compaction

## Abstract

The NKX2-5 gene encodes for a transcription factor crucial for cardiac cell differentiation and proliferation. It was the first gene associated with congenital heart disease (CHD) in humans and has been linked to conduction disorders or cardiomyopathies. However, an overlapping phenotype is not frequent in the literature. We describe a family with a novel missense mutation in the NKX2-5 gene (p.Gln181Pro) with numerous antecedents with atrial septal defect (ASD), left ventricular non-compaction (LVNC), conduction disease, and sudden cardiac death (SCD).

## Introduction

The NKX2-5 gene is located on chromosome 5, spanning two exons and encoding 324 amino acids (1). It belongs to the NK homeobox gene family and encodes for a transcription factor with three domains involved in cardiac development. The homeodomain is located at amino acid positions 138–197 and is required for interaction with DNA and other transcription factors (1). A few genetic variants in this region have been associated with the development of atrial septal defects (ASD), cardiomyopathies, and conduction defects, with variable penetrance and expressiveness, most of them with evidence of cosegregation.

We present a family with a previously unreported mutation in NKX2-5 and phenotype overlap between congenital heart disease (CHD), conduction disease, and non-compaction cardiomyopathy, with some cases of sudden cardiac death (SCD).

## Case Description

**Figure 1:**
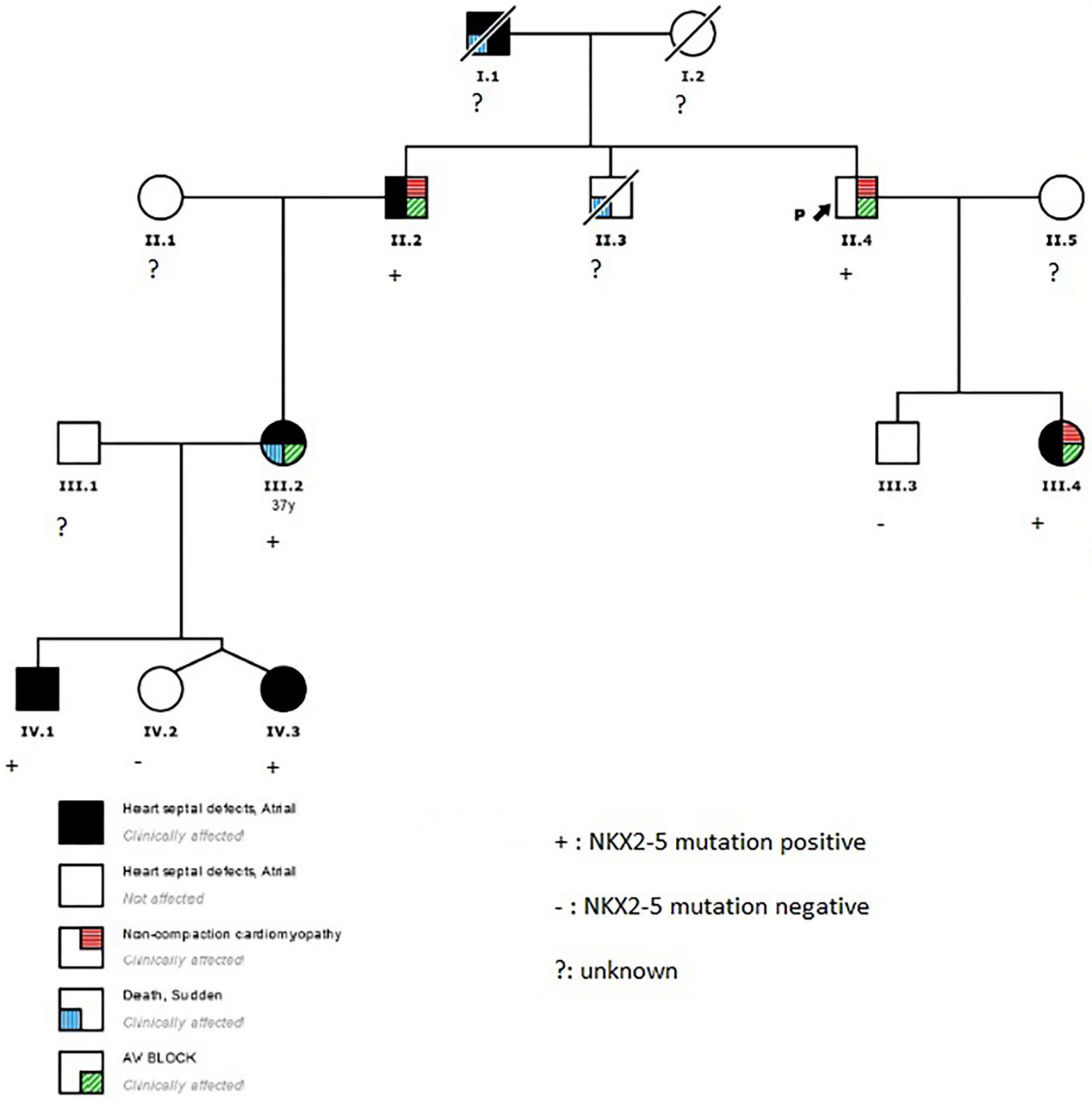
Family pedigree. Black arrow, proband; square, male; circle, female; black, heart septal defects; white, not affected; diagonal stripe, death; red lines, non-compaction cardiomyopathy; blue lines, sudden death; green lines, atrioventricular block.

**Figure 2:**
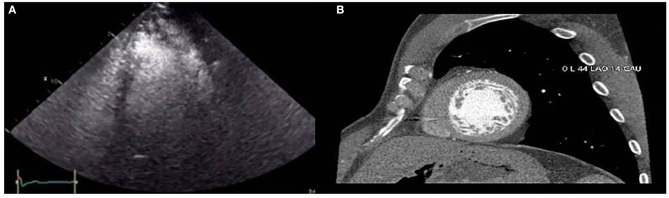
**(A)** Contrast echocardiography showing left ventricular non-compaction. **(B)** Hypertrabeculated left ventricle in one slice of the short axis view by coronary computed tomography angiography.

The proband (II.4 in Figure 1) who belongs to a family of European ancestry has numerous descendants with ASD and sudden death of his father at 48 years and one brother at 43 years old (Figure 1 and Table 1). At 42 years of age, he had a pacemaker implanted due to a finding of asymptomatic complete atrioventricular (AV) block. The echocardiogram showed a 40% left ventricular ejection fraction, and coronary heart disease was ruled out with coronary computed tomography angiography (CCTA) and catheterization. An echocardiogram with contrast was performed as his device was not compatible with cardiac magnetic resonance (CMR). This test showed a hypertrabeculated myocardium of the left ventricle, also present in the previous CCTA performed (Figure 2) and compatible with left ventricular non-compaction (LVNC) with a ratio of non-compacted/compacted myocardium of 2.2. In the proband's device, paroxysmal atrial fibrillation and non-sustained ventricular tachycardia (NSVT) were recorded. Beta-blocker and angiotensin-converting enzyme inhibitor were prescribed, and we decided to upgrade to implantable cardioverter-defibrillator (ICD) and cardiac resynchronization therapy.

**Table 1:** Clinical phenotype of family members.

**Family member**	**Genotype**	**Age at time of study**	**Sudden death (years)**	**Documented tachyarrhythmias**	**Conduction disease**	**LVNC**	**LVEF (%)**	**CHD**
I.1	Not done	Deceased	Yes (48)	No	None known	No	58	ASD
II.2	+	61	No	NSVT	Junctional rhythm	Yes	43	ASD
II.3	Not done	Deceased	No (43)	No	None known	None known	None known	None known
II.4	+	42	No	NSVT	Third-degree AV block	Yes	40	No
III.2	+	37	Yes (37)	VF	First and third degree AV block	No	60	ASD VSD
III.3	–	28	No	No	No	No	59	No
III.4	+	19	No	No	First-degree AV block Second-degree AV block	Yes	58	ASD
IV.1	+	1	No	No	No	No	65	ASD VSD PLSVC Absent right SVC
IV.2	–	1	No	No	No	No	64	No
IV.3	+	1	No	No	No	No	66	ASD

*ASD, atrial septal defect; AV, atrioventricular; CHD, congenital heart diseases; LVNC, left ventricular non-compaction; LVEF, left ventricular ejection fraction; NSVT, non-sustained ventricular tachycardia; PLSVC, persistent left superior vena cava; SVC, superior vena cava; VF, ventrtricular fibrillation; VSD, ventricular septal defect*.

The proband's niece (III.2 in Figure 1) had a history of surgical patch closure of ASD and perimembranous ventricular septal defect (VSD). She also had a daughter with ASD (IV.3) and a child (IV.1) with ASD, VSD, persistent left superior vena cava (SVC), and absent right SVC. The echocardiogram during follow-up was normal, and in a previous electrocardiogram (ECG) she presented first-degree AV block, negative T waves in precordial leads, and incomplete right bundle branch block. At 37 years old, she survived a sudden cardiac arrest secondary to extreme bradycardia at presentation that seemed to be a third-degree AV block; it quickly degenerated into ventricular fibrillation. After cardiac arrest, a new echocardiogram was performed that did not show anomalies. She elected for ICD implantation and next-generation sequencing gene panel for cardiac conditions.

The proband's brother (II.2 in Figure 1) had a history of surgically repaired ASD and nodal rhythm. He was diagnosed by CMR with a mildly reduced ejection fraction and multiple prominent ventricular trabeculations with a ratio of non-compacted/compacted myocardium of 2.4 compatible with LVNC. A 24-h ECG Holter was performed that documented atrial fibrillation and non-sustained polymorphic ventricular tachycardia. He was in a New York Heart Association (NYHA) II class, and the exercise test showed non-sustained polymorphic and monomorphic ventricular tachycardia. Based on these findings, beta-blocker, angiotensin-converting enzyme inhibitor, and antialdosteronic drugs were prescribed, and he was elected to undergo prophylactic ICD implantation.

**Figure 3:**
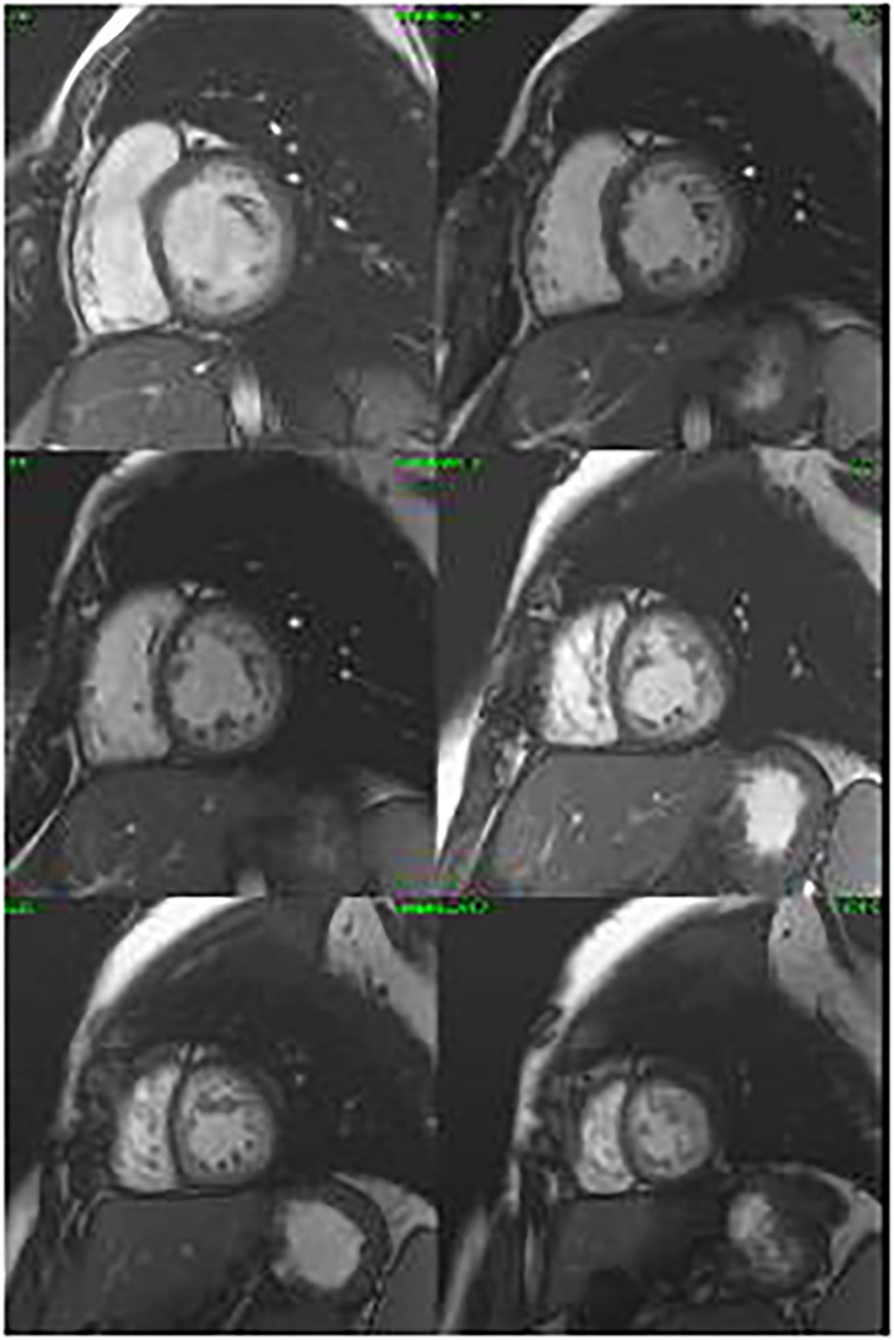
Cine cardiac magnetic resonance images in the short axis revealing non-compaction cardiomyopathy.

The proband's daughter had a history of surgically repaired ASD. She was studied in our unit at the age of 20. She was completely asymptomatic. A 12-lead ECG showed first-degree heart block and ST depression in the lateral leads. The CMR confirmed a non-compacted myocardium in three segments, compatible with LVNC (Figure 3). The volumes were in the normal range, and the left ventricular ejection fraction was 58%. The exercise test was normal, and we performed a 24-h ECG Holter that recorded first-degree AV block and Mobitz type 1, without significant arrhythmias. An implantable loop recorder was inserted, and there were no ventricular arrhythmias or advanced AV block reported.

Genetic testing of the proband's niece using next-generation sequencing showed only a novel missense mutation in the NKX2-5 gene (NKX2-5 NM_004387.4; c.542A>C; p.Gln181Pro; GRCh37/hg19: Chr5; g.172660005), probably related to the phenotype. The rest of the family members (II.2, II.4, III.3, III.4, IV.1, IV.2, and IV.3) were screened for the variant. All the living affected individuals tested positive for the variant, while individuals tested with normal cardiac function were negative for the mutation.

## Discussion

The NKX2-5 gene plays an important role in heart formation and development. Some studies have evaluated mutations in the NKX2-5 gene being more frequent in Europe (24.1%) and less in Africa (1.49%). The c.325G>T (62.5%) and c.896A>G (52.9%) had the most frequency (2).

A pathogenic genetic variant in NKX2-5 was the first found to be associated with CHDs (3). Gade et al. screened 39 CHD families for NKX2-5 mutations. Mutations were significantly more common in familial cases than non-familial cases (*p* < 0.001) (4). Likewise, genetic variants in NXK2-5 were also identified as the factors responsible for VSDs, patent ductus arteriosus, and the tetralogy of Fallot (5).

NKX2-5 has been found to be linked to AV conduction blocks due to its role in the regulation of electrophysiological properties (6). In humans, the most commonly reported phenotypes are CHD and AV conduction disease. Perera et al. described a missense mutation (pGln181His) in 11 phenotypically affected family members with a strong family history of CHD, AV block, and SCD (7). Mutations in the NKX2-5 gene have also been reported in patients with dilated cardiomyopathy and LVNC (8, 9). In a study by Yuan et al., dilated cardiomyopathy was documented in a family with a novel heterozygous NKX2-5 mutation (p.Ser146Trp). Mutation carriers also had AV block and arrhythmias like atrial fibrillation (8). SCD has been described and is generally attributed to progressive conduction disease and ventricular arrhythmias (10); however, SCD has been reported in patients with a normally functioning pacemaker and absence of cardiomyopathy (7).

The relevant finding of this study is the identification of a novel missense genetic variant in NKX2-5 (p.Gln181Pro), which is absent in the general population. This variant has been tested by 21 “*in silico*” prediction programs and was pathogenic in all of them. It belongs to a hot-spot region with several pathogenic variants and was not found in gnomAD exomes or genomes. All of the above and the cosegregation of variant and phenotype could be in favor of its pathogenicity. Nevertheless, not all family members underwent a genetic study, and the majority were only screened for the variant. Therefore, mutations in other genes could not be ruled out.

In conclusion genetic testing should be considered in familial cases of CHD, conduction disease, and LVNC. The identification of these variants has important diagnostic and management implications for the extended family, largely due to the risk of individuals with NKX2-5 mutations to present SCD. We suggest that the risk of lethal arrhythmias and conduction disorders, as well as cardiac morphology and function, should be evaluated routinely in order to assess the need for ICD or pacemaker implantation.

## Data Availability Statement

The original contributions presented in the study are included in the article/supplementary materials, further inquiries can be directed to the corresponding author/s.

## Ethics Statement

Written informed consent was obtained from the individual(s) for the publication of any potentially identifiable images or data included in this article.

## Author Contributions

All authors contributed in writing the manuscript and approved the submitted version.

## Conflict of Interest

The authors declare that the research was conducted in the absence of any commercial or financial relationships that could be construed as a potential conflict of interest.
